# Structures and Ribosomal Interaction of Ribosome-Inactivating Proteins

**DOI:** 10.3390/molecules21111588

**Published:** 2016-11-21

**Authors:** Wei-Wei Shi, Amanda Nga-Sze Mak, Kam-Bo Wong, Pang-Chui Shaw

**Affiliations:** Centre for Protein Science and Crystallography, School of Life Sciences, The Chinese University of Hong Kong, Shatin, N.T., Hong Kong, China; Shiww@cuhk.edu.hk (W.-W.S.); amandamak@gmail.com (A.N.-S.M.); kbwong@cuhk.edu.hk (K.-B.W.)

**Keywords:** ribosome-inactivating proteins, RNA *N*-glycosidases, ribosome, P proteins, ribosomal interaction

## Abstract

Ribosome-inactivating proteins (RIPs) including ricin, Shiga toxin, and trichosanthin, are RNA *N*-glycosidases that depurinate a specific adenine residue (A-4324 in rat 28S ribosomal RNA, rRNA) in the conserved α-sarcin/ricin loop (α-SRL) of rRNA. RIPs are grouped into three types according to the number of subunits and the organization of the precursor sequences. RIPs are two-domain proteins, with the active site located in the cleft between the N- and C-terminal domains. It has been found that the basic surface residues of the RIPs promote rapid and specific targeting to the ribosome and a number of RIPs have been shown to interact with the C-terminal regions of the P proteins of the ribosome. At present, the structural basis for the interaction of trichosanthin and ricin-A chain toward P2 peptide is known. This review surveys the structural features of the representative RIPs and discusses how they approach and interact with the ribosome.

## 1. Introduction

RNA *N*-glycosidases are very potent enzymes, and some of them are among the most potent toxins of plant origin. Ricin, one of the most toxic natural substances discovered in late 19th century by P. H. Stillmark, agglutinates red blood cells [1] and its lethal dose in humans is about 1.78 mg for an average adult [2]. It is now known that ricin hydrolyzes the *N*-glycosidic bond at adenine 4324 (A-4324) in the 28S ribosomal RNA (rRNA) of eukaryotic ribosomes [3], and in some cases A-2660 in the naked 23S rRNA of prokaryotic ribosomes [4]. This adenine is located at a GAGA hairpin within the α-sarcin/ricin loop (α-SRL) [5]. The loop is highly conserved in all large ribosomal subunits and is essential for the proper assembly of the functional core of the large subunit [6]. In eukaryotes, removal of the specific adenine hinders the elongation factor 1-dependent binding of aminoacyl-transfer RNA (tRNA) and guanosine triphosphate (GTP)-dependent binding of elongation factor 2 to ribosome. In prokaryotes, damaged ribosomes do not bind elongation factor thermo unstable (EF-Tu) or elongation factor (EF) G G. As a result, protein synthesis is arrested at the elongation step [7]. rRNA *N*-glycosidases are therefore also known as ribosome-inactivating proteins (RIPs). There are also other classes of proteins that inactivate ribosomes. These include phosphodiesterases such as α-sarcin and restrictocin that inactivate ribosome through the hydrolysis of a single phosphodiester bond between G4325 and A4326 in the α-SRL [8] and adenosine diphosphate (ADP)-ribosyltransferase such as diphtheria toxin that catalyzes the transfer of the ADP-ribose of niconiteamide adenine nucleotide (NAD) to the diphthamide moiety of EF-2 [9]. However, it is a common practice to reserve the name RIP to RNA *N*-glycosidases and these two terms are used interchangeably here.

Under physiological conditions, a single ricin A chain (RTA) molecule depurinates 1000–2000 mammalian ribosomes per minute. Naked rRNA is less susceptible to RIPs, indicating that RIPs need to bind to specific ribosomal proteins before carrying out their catalytic action [10]. Besides the RNA *N*-glycosidase activity, RIPs are also found to possess specific DNA cleavage activities on double-stranded supercoiled DNA and mitochondrial DNA [11], superoxide dismutase [12], phospholipase [13], or DNA topoisomerase inhibitory activities [14], although it is possible that some of these activities may be due to contamination in protein preparation [15]. With more than 20 RIP structures available and the recent discoveries on the RIP-ribosome interaction, we set to provide a survey on the structures of RIPs and their interaction mode with the ribosomes.

## 2. Distribution and Classification of RIPs

RIPs are widely distributed in over 100 different plant species and in different organs [16,17]. They are also found in fungi, algae, and bacteria [18,19]. However, some of these RIPs have very different molecular weights and N-terminal protein sequences and may not be true RNA *N*-glycosidases. RIPs might play an important role in plant physiology and defense mechanisms because some of them could be induced by development [20], stress [21], or viral infection [22,23].

**Figure 1:**
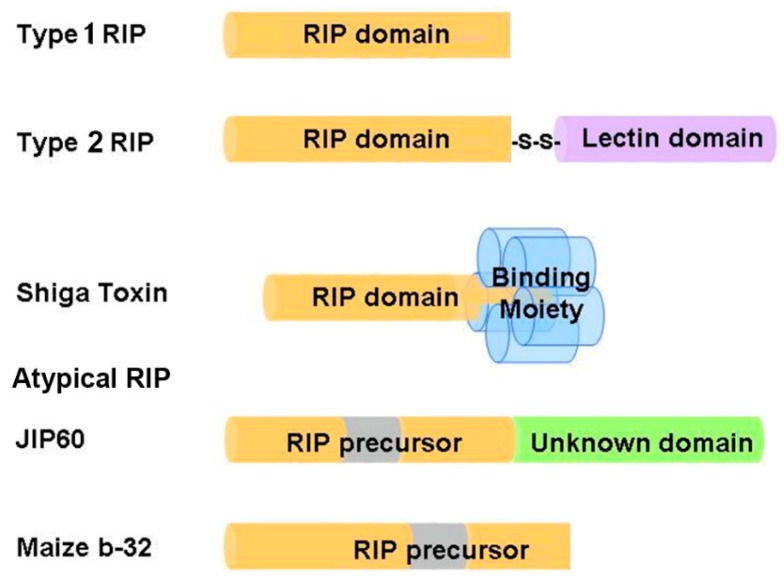
Schematic representation of the organization structures of ribosome-inactivating proteins (RIPs). The RIP domains are colored yellow; lectin domain is colored purple; binding moiety of Shiga toxin is colored blue; unknown domain is colored green; internal inactivation fragments being removed during maturation are colored gray. JIP60 = jasmonate-induced protein.

Based on the number of subunits and the organization of the precursor sequences, RIPs are grouped into three types (Figure 1). Type 1 RIPs such as trichosanthin (TCS), pokeweed antiviral protein (PAP) and saporin (SO6) consist of a single polypeptide, with molecular weight around 28 kDa. Type 2 RIPs such as ricin and abrin consist of two polypeptide chains linked by a disulfide bridge, with molecular weight around 60–65 kDa. Chain A is the catalytic subunit that is homologous to type 1 RIPs, while chain B is a lectin domain that facilitates the intracellular delivery of chain A by interacting with glycoconjugates on the cell surface [4]. As a result, type 2 RIPs are in general more cytotoxic than type 1 RIPs. Some type 2 RIPs such as eublin and cinnomomin are less toxic because the sugar-binding amino acid residues in the lectin domain have impaired affinity for galactosides, resulting in a greatly reduced uptake of these RIPs [24]. Shiga toxin is a representative bacterial RIP, comprising a catalytic A subunit (30 kDa) and five B subunits (7 kDa) that bind specifically to the glycolipid receptor on cell surface [25]. Atypical RIPs or type 3 RIPs such as maize b-32 and jasmonate-induced protein (JIP60) have unusual precursor sequence organization (Figure 1). Maize b-32 is synthesized from a large proenzyme [16]. During germination, the N-terminal and C-terminal pro-sequences and an internal fragment of the proenzyme precursor are removed by proteolysis to generate an active and basic protein of 248 residues (MOD) [26]. After removal of the internal fragment, the activity of MOD is at least 600-fold higher than immature maize RIPs [27]. JIP60 consists of an RIP domain and an unknown domain at the C-terminal and is activated upon deletion of the C-terminal domain and an internal fragment in the RIP domain [28]. As the Pfam protein families database [29] summarized, RIP domain not only has been found duplicated or triplicated in a protein, but it can also fuse with variant functional domains, such as peptidase C48, Uridine 5′-diphospho-glucuronosyltransferase, amino acid kinase, RNA recognition motif, reverse transcriptase, glycoside hydrolase family 18 domain etc. These additional domains may impart distinct functions to RIPs.

## 3. Crystal Structure and Structural Comparison of RIPs

**Figure 2:**
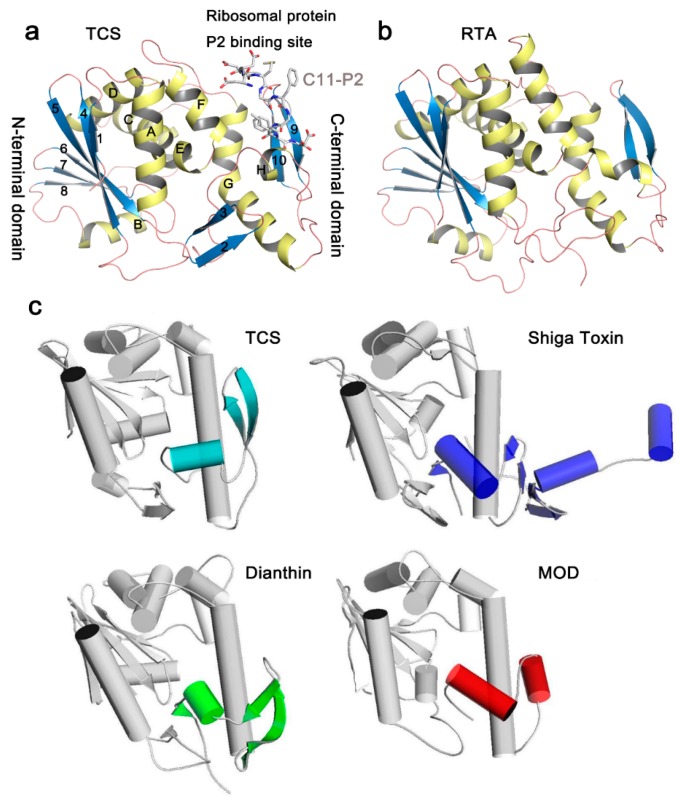
Representative structures and fold of RIPs. (**a**) Structure of TCS complexed with a C-terminal 11aa fragment of the ribosomal P protein (PDB code: 2JDL). The conserved secondary elements are labeled individually. α-Helices are colored yellow, β-strands are colored blue and loops are colored pink. The last 11 residues of ribosomal stalk protein P2 (C11-P2) are shown as gray sticks; (**b**) The structure of the catalytic chain A of Ricin (RTA); (**c**) Structural comparison of selected RIPs with TCS. The C-terminal domain of TCS is colored light blue. Differences in the structural features compared to TCS are highlighted in the RIP. It is found that the N-terminal domains are more conserved among the RIPs.

**Figure 3:**
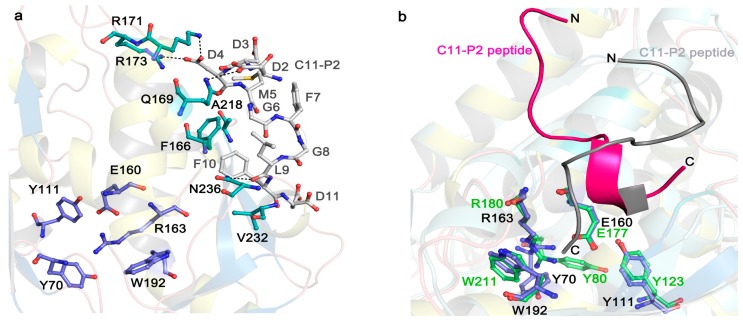
Stereo image on the active site and P2 binding mode of TCS (PDB code: 2JDL) and RTA (PDB code: 5GU4). (**a**) The active site and P2 binding pocket of TCS. The conserved active site residues in TCS are shown in purple sticks. The P2 binding residues are shown in cyan sticks. The C11-P2 peptide is shown as gray sticks. Hydrogen bonds are highlighted with black dash lines; (**b**) P2 peptide adopts distinct conformation for binding to TCS and RTA. The conserved active site residues in RTA are shown in green sticks. The magenta and gray colored C11-P2 peptides indicate the orientations of P2 peptide in binding to RTA and TCS, respectively.

In the Protein Data Bank (PDB), structures of more than 20 RIPs are available (Table 1). Although the amino acid sequence identity in RIPs is less than 50% and antibodies raised against one RIP usually do not cross-react with one another [16], the key residues of *N*-glycosidase catalytic subunit of RIPs are highly conserved. In general, the *N*-glycosidase subunit of RIPs contains two domains, the large N-terminal domain consists of six α-helices and a six-stranded mixed β-sheet, while the small C-terminal domain consists of an anti-parallel β-sheet and an α-helix with a bend in the middle (α-helix G and H) (Figure 2a). The invariant active site residues Y70, Y111, E160, R163, and W192 (reference to TCS sequence in PDB code 2JDL) are located in the cleft between the N-terminal and C-terminal domains (Figure 3a). At the C-terminal region, the hydrogen bonds L240-*N*-P35-*O* and L240-*O*-L37-*N* in TCS are highly conserved among the known structures. These hydrogen bonds are essential to maintain structure stability between the N- and C-terminal domains, and deletion of them has been shown to disrupt the folding of TCS [30]. The ribosomal protein binding site is located between the anti-parallel beta-sheets 9 and 10 in the C-terminal domain [31].

The structures of RIPs are, in general, well-conserved. For example, the overall root-mean-square deviation (RMSD) of TCS and RTA is 1.1 Å (Table 1). Between TCS and RTA, the major differences occur in the N-terminal domain, in which strands 2 and 3 are missing (Figure 2b). These two strands are far away from the active site. We have also compared the structures of RIPs that have RMSD > 2 with TCS (Figure 2c). In saporin, β-strands 9 and 10 in the C-terminal domain are replaced by a short loop. Dianthin does not have strand 2. It has a long loop between strands 6 and 7 and a dissimilar C-terminal region. The structures of Shiga toxin and maize RIP are more deviated from TCS. In Shiga toxin, the C-terminal domain only reserves the bended α-helices but has two extra α-helices and four-stranded mixed β-sheets. For the mature form of the maize RIP, the α-helix B and β-strand 8 in the large domain are missing and the anti-parallel β-strands 9 and 10 in the small domain are replaced by a short α-helix. As shown in the structural comparison, the small domains are, in general, more varied. As discussed below, the small domains in several RIPs are shown to bind ribosomal P proteins. Structural variation of this domain may influence the specificity to the target ribosomal subunit.

In type 2 RIPs, the *N*-glycosidase domain (chain A) is linked with a lectin domain (chain B) by a disulfide bond. The chain B binds to β-1,4-linked galactose residues on the cell surface and facilitates delivery of chain A. The overall structure of ricin chain B (RTB) is highly conserved and consists of two homologous domains arisen by gene duplication. Each domain is composed of 12 β-strands and is arranged in a β-trefoil structure of three lobes α, β, and γ [33]. The sugar-binding pockets are located in 1α and 2γ sub-domains in RTB, and consist of aspartic acid, valine, arginine and a variable aromatic residue to provide a sugar binding platform [34].

## 4. Invariant Residues in the Catalytic Subunit

**Figure 4:**
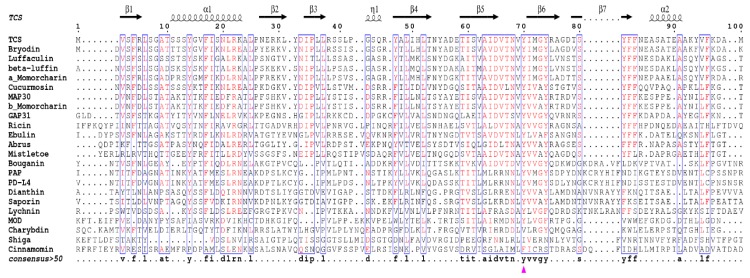

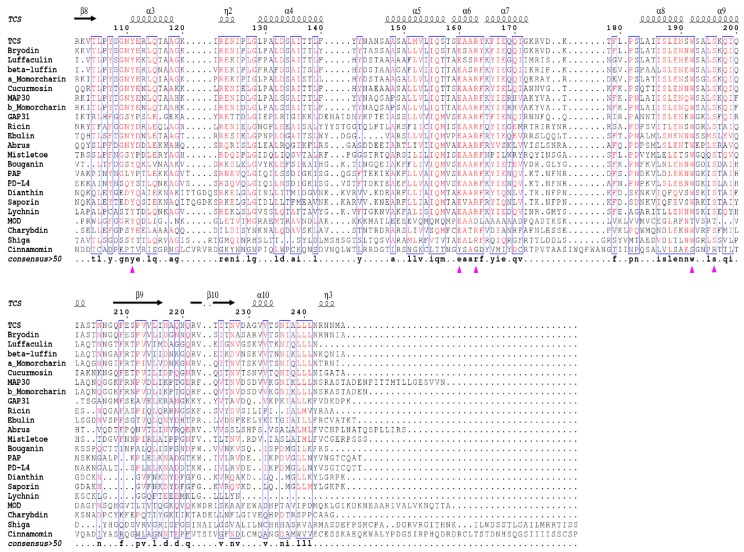
Structure based sequence alignment of structurally known RIPs. The catalytic A chains of all type 2 RIPs were used for sequence alignment. The sequence alignment was prepared by ENDscript 2 web server [40]. The conserved key residues of the active pocket of TCS (Y70, Y111, E160, R163, and W192) are marked with purple triangles. The top secondary structure elements are shown according to the crystal structure of TCS. The consensus sequences are highlighted with blue rectangles (consensus value >50%), the highly conserved residues are colored in red.

Sequence alignment of all these structure available RIPs indicates the existence of invariant residues, including Y14, R22, Y70, Y111, E160, R163, W192, and S196 as reference to TCS (Figure 4). Although Y14 and R22 are invariant, they are not crucial for the activity [35]. On the other hand, the active site residues Y70, Y111, E160, R163, and W192 are structurally conserved (Figure 3a). The orientation of the active site Y70 tyrosine ring is flexible and this may facilitate substrate binding. W192 lies at the bottom of the active site pocket, defines the binding site, stabilizes the ligand inside the cavity, and protects it from the solvent. The indole ring shows hydrophobic interaction with R163 and L241, which is essential for structure stability. S196 is located within α-helix H near the active site and its side chain forms two hydrogen bonds with both backbone and side chain of active site W192, and is important for holding the indole ring and stabilize adenine binding. The mechanism of catalysis has been described previously [36].

In many RIPs, there are two key glutamate residues in the active site. In TCS, they are E160 and E189. The active site residue E160 serves to stabilize the ribooxocarbenium ion-like transition state intermediate, while E189 acts as back-up for the catalytic glutamate in case the latter is mutated [37,38]. However, maize RIP, Shiga toxin, and known RIPs of the Family Poaceae have only one glutamate in the active site. Structure-function study of maize RIP indicates the active site pocket of maize RIP is too small for two glutamate residues and it is suggested that maize RIP may be evolutionarily more related to bacterial RIPs [39].

## 5. The Interaction of RIPs with Ribosomes

**Figure 5:**
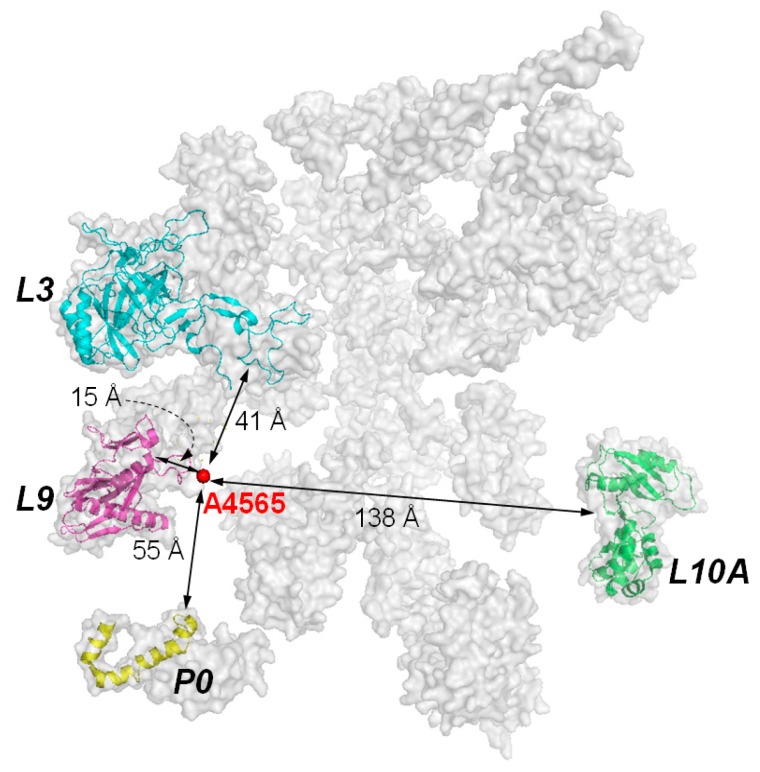
Ribosomal subunits that have been found to interact with RIPs. Subunits P0, L3, and L9 are clustered on a plane and within the vicinity of the targeted adenine. Subunit L10A is almost at the opposite side of the other three subunits. The interaction with the former three ribosomal subunits may be more relevant to the biological activity of RIPs, as variants of TCS with reduced interaction with P proteins are less active in ribosome-inactivation. Model of mammalian ribosome is according to PDB, code 2ZKR.

Although the target site of RIPs is a specific adenine residue in the 28S RNA, the presence of ribosomal proteins is essential for efficient catalysis. It was observed that the k_cat_ value of RTA on naked RNA is 10^5^-fold slower than on intact ribosome [10]. In PAP, the binding affinity on naked rRNA is 10-fold weaker than on intact ribosome [41]. Subsequently, RIPs have been shown binding to specific ribosomal proteins. For examples, TCS binds to the acidic ribosomal P proteins and L10a [31,42]; RTA binds to ribosomal protein L9 and P0 (=L10e) [43]; and PAP binds to L3 [41,44,45]. RTA and Shiga-like toxin 1 bind to the C-terminal region of P protein [46] and depurination of ribosome is reduced when RTA is expressed in the *Saccharomyces cerevisiae* mutants with P1 or P2 deleted. These mutants are more resistant to the cytotoxicity of RTA [47]. These indicate that the ribosomal stalk facilitates the recruitment of some RIPs by transporting them to the spatial proximity of SRL. The known ribosomal proteins which were identified to interact with RIPs and their distribution around the target adenine of the α-SRL are shown in Figure 5.

The early RTA and ribosome interaction studies showed that the electrostatic surfaces of RTA and ribosome are essential for the delivery of RTA to the surface of ribosome [48,49,50]. The target location may be achieved through the following steps: (i) the toxin is oriented for productive association and catalysis when it approaches the ribosome; (ii) the toxin is attracted to the ribosome even if they are far apart; and (iii) once the toxin binds to the ribosome, it is guided to the specific ribosomal subunit by the electrostatic field on the ribosome, probably through several association-dissociation processes [48,49,50]. The subsequent proposed two-step interaction model stated that the initial non-specific electrostatic interaction increases local concentration of RTA, facilitating the encounter and accelerating the reaction rate above the expected diffusion limit [51]. Then the more specific interaction of RTA with the ribosomal stalk pentamer facilitates the proximity to the α-SRL of ribosomes [51,52]. This two-step model was confirmed by the kinetic observations that the interaction of RTA with intact pentameric ribosomal stalk fit well with a simple 1:1 interaction model, and the association rate constant of the RTA-intact stalk pentamer interaction was two-fold greater than its association rate with the stalk trimers, which contain only one P1/P2 heterodimer [53]. These results indicating multiple copies of the stalk proteins or intact ribosomal stalk pentamer can accelerate the recruitment of RTA to ribosome for depurination [52,53].

Recent structural studies have revealed how eukaryotic stalk protein recruits TCS or RTA to ribosomes. The crystal structure of TCS and P protein C-terminal peptide complex [54] shows that three previously identified positively charged residues, K173, R174, and K177 [31,55] form favorable electrostatic interactions with the P protein peptide. Besides F166 and V232 (as shown in Figure 3a), other residues A184, L188, L215, and I225 around 4 Å from P2 peptide may form a hydrophobic pocket for the interaction. Crystal structure of RTA-P2 peptide reveals that the binding manner of RTA and P protein peptide is similar to that with TCS. RTA donated a unique hydrophobic pocket to stabilize the C-terminal hydrophobic GFGLFD motif of P2 peptide, while the structurally untraced acidic SDDDM motif of P2 peptide was shown by biochemical interaction assays for charge-charge interaction with RTA (Figure 3b) [56]. The structural superposition of the TCS-P2 and RTA-P2 complexes demonstrated P2 peptide adopted distinct orientations and different interaction modes while binding to these two RIPs (Figure 3b). In SO6, K220, K226, and K234 in the C-terminal domain are protected by ribosome upon differential succinic anhydride modification [55]. Interestingly, these charged residues in SO6 are located in a region similar to that of TCS, indicating that the two proteins may use the similar site to interact with ribosome. Docking analyses showed that SO6 and Shiga toxin may interact with P proteins in a manner similar to TCS, of which there is a charge-charge interaction at the N-terminal region of the P peptide and hydrophobic interaction at the C-terminal region [54,57]. In the case of TCS and ribosome interaction, the structure of the full-length human P1/P2 revealed the well-folded N-terminal dimerization domain and a C-terminal domain, linked by a proline-alaline rich linker that can extend C-terminal tail up to 125 Å [58]. The long flexible linker presumably plays an important role in reaching out to capture the elongation factors nearby [59,60]. Truncation of the linker region results in greatly reducing the depurination activity [58]. These observations suggest that the flexible linker may sweep around to recruit RIPs that are attracted by the ribosome and deposit them to the specific adenine on the α-SRL. It has also been shown that the C-terminal tail and flexible linker of the ribosomal stalk are essential for binding the eukaryotic factors 2 (eEF2) [59,61,62]. After binding to eEF2, ribosomes can be protected from RIP depurination [63,64], suggesting that RIPs and eEF2 may compete for binding to the ribosomal stalk. Therefore, eukaryote-specific RIPs may hijack the elongation-factor recruiting function of ribosomal stalk in reaching the α-SRL [57].

However, this approach of interaction may not be universal. Comparison of primary sequences shows that the residues located at the C-terminal domain, which is responsible for P protein interaction in RIPs, are not conserved (Figure 4). Besides, for the PAP-ribosome interaction, N69, F90, N91, and D92 in the active site cleft of PAP are shown to be important for binding with L3 [41] and the C-terminal region of the P protein is not required for PAP to get access to the ribosome [65]. In maize RIP, the corresponding anti-parallel beta-sheets are replaced by a short α-helix, and no positively charged residues are found. It has been shown that MOD, the active form of maize RIP, interacts with the conserved C-terminal peptide of P2 without hydrophobic interactions [66]. Four positively charged lysines K143-K146 in MOD were identified to be involved in interacting with the negatively charged DDD motif on P2 [67]. These positively charged amino acids on MOD are located at the base of the internal inactivating loop, which is removed for the activation of maize RIP.

## 6. Kingdom Specificity of RIPs to Ribosome

Apart from acting on eukaryotic ribosomes, PAP, dianthin, mirabilis antiviral protein, and Shiga toxin also capable of depurinating prokaryotic ribosomes at A2660 in 23S rRNA, the equivalent adenine in eukaryotic 28S rRNA. The efficiency to prokaryotic ribosomes is about 100–500 times weaker [68] or comparable to eukaryotic ribosomes [69]. The target adenine in GAGA hairpin within the α-SRL is conserved in most bacteria, plants, and animals. Some RIPs such as RTA do not act on prokaryotic ribosome but can depurinate naked 23S rRNA [10]. Since the active site residues are invariant in all RIPs, it is possible that the ribosomal proteins play a role to determine the kingdom specificity of RIPs. For example, PAP was found to interact with L3, which is highly conserved in yeast, human, and E. coli, hence the access of ribosome through L3 may justify its dual specificity on eukaryotes and prokaryotes [47].

## 7. Conclusions

RIPs are potent toxins. On one hand, they may be used as bioweapons or cause food poisoning. On the other hand, they have good potential applications in pharmacology and biotechnology [70,71,72]. The RIP conjugated immunotoxins have exhibited promising tumor inhibition for clinical use [73,74]. For examples, SO6 and RTA immunotoxins can effectively kill lymphocytes in allograft-related diseases [75,76]. Besides, Shiga toxin can remove contaminated tumor cells in stem cell grafts [77]. TCS has been tested as anti- human immunodeficiency virus (HIV) agent [78] and used to induce mid-term abortion [36]. PAP and maize RIP are promising anti-insect and anti-fungal agents in transgenic plants [39,79,80]. Through fusing the transduction domains to type 1 RIP dianthin [81,82] or saporin [83,84], their intracellular routing can be altered for enhanced cytotoxicity and tumor inhibition. Besides, we have shown that the active form of maize RIP (MOD) protect chimeric simian-human immunodeficiency virus-infected macaque peripheral blood mononuclear cells from lysis ex vivo and transiently reduce plasma viral load in a simian-human immunodeficiency virus (SHIV) 89.6-infected rhesus macaque model [85]. The specificity of maize RIP to HIV-infected cells can be increased by fusing the HIV transduction peptide to the N-terminal of maize RIP and by introducing HIV-1 protease recognition sequences to the internal inactivation region of this protein [85,86]. With the potential uses of ribosome-inactivating proteins in different areas, it is important to understand how they inactivate ribosome and cause cell death. The evidence gathered so far indicates that ribosome binding is crucial for kingdom specificity and ribosome-inactivating activity of RIPs. On the ribosome, the P protein complex close to the α-SRL, may offer a major site for RIP to bind and it is found that a common surface on some RIPs takes part in the interaction with the C-terminal region of P protein. Nevertheless, there remain some unsolved questions: (i) Some RIPs may bind to other ribosomal subunits. What is the significance of this binding and how many sites on the ribosome are available for RIPs to dock? (ii) Why some RIPs possess dual specificity? (iii) After docking on the ribosomal protein, how does the RIP find the target adenine? Further work on the ribosome recognition is required.

## Figures and Tables

**Table 1:** Available structures of representative RIPs and their similarity in comparison with trichosanthin (TCS).

RIP	Source	Ligand	PDB	Resolution (Å)	Cα RMSD
Type 1					
Bouganin	*Bougainvillea spectabilis*	-	3CTK	1.8	1.1
Bryodin 1	*Bryonia dioica*	-	1BRY	2.1	0.4
Charybdin	*Charybdis maritima*	2-*N*-morpholino-ethanesulfonic acid	2B7U	1.6	1.9
Cucurmosin	*Cucurbita moschata*	-	3BWH	1.0	0.4
Dianthin	*Dianthus caryophyllus*	Adenine	1LPD	1.7	2.6
Luffaculin 1	*Luffa acutangula*	*N*-Acetyl d-glucosamine	2OQA	1.4	0.5
beta-luffin	*Luffa cylindrica*	*N*-Acetyl-d-glucosamine	1NIO	2	0.5
GAP31	*Gelonium multiforum*		3KTZ	1.6	0.745
Lychnin	*Lychnis chalcedonica*	-	2G5X	1.7	1.9
MAP30	*Momordica charantia*	-	1D8V	NMR	1.6
α-Momorcharin	*Momordica charantia*	Adenine	1AHA	2.2	0.5
β-Momorcharin	*Momordica charantia*	Modified hexasaccharide	1CF5	2.6	0.6
PAP	*Phytolacca americana*	2-(acetylamino)-2-deoxy-A-d-glucopyranose	1GIK	1.8	1.6
PD-L4	*Phytolacca dioica*		2Z4U	1.1	1.7
TCS	*Trichosanthes kirilowii*	Adenine	2JDL	1.8	-
Saporin	*Saponaria officinalis*	-	1QI7	2	2.0
Type 2					
Abrus agglutinin 1	*Abrus precatorius*	*N*-Acetyl-d-glucosamine	2Q3N	3.5	1.0
Cinnamomin	*Cinnamonum camphora*	-	2VLC	2.95	0.752
Ebulin	*Sambucus ebulus*	Beta-d-galactose	1HWM	2.8	0.9
Mistletoe lectin 1	*Viscum album*	*N*-Acetyl-d-glucosamine	1ONK	2.1	1.0
Ricin	*Ricinus communis*	Adenine	1IFS	2	1.1
Shiga toxin	*Bacteriophage 933W*	Adenine	2GA4	1.8	2.9
Atypical RIP					
MOD	*Zea mays*	-	2PQI	2.5	3.4
Maize RIP	*Zea mays*		2PQG	2.4	3.8

Cα RMSD (Root mean square deviation) with reference to TCS was calculated by PyMOL (De Lano Scientific, San Carlos, CA, USA) [32]. All the structures were solved by X-ray crystallography, except MAP30, which was solved by NMR. PDB = Protein Data bank; GAP 31 = Gelonium Anti-HIV Protein, MW 31 kDa; MAP30 = (Momordica Anti-HIV Protein, MW 30 KDa; PAP = pokeweed anti-viral protein; PD-L4 = The RIP from leaves of *Phytolacca dioica*; MOD = the active form of maize RIP.
